# Differential Temporal Dynamics and Motor–Non-Motor Dissociation After Inhaled Levodopa in Parkinson’s Disease: A Post Hoc Analysis of the INLEVO-LIFE PD Study

**DOI:** 10.3390/medsci14060680

**Published:** 2026-10-17

**Authors:** Diego Santos García, María Cerdán Sánchez, Rosa Yáñez Baña, Iria Cabo, Maria Icíar Cimas Hernando

**Affiliations:** 1 Department of Neurology, Complejo Hospitalario Universitario de A Coruña (CHUAC), 15006 A Coruña, Spain; 2 Grupo de Investigación en Enfermedad de Parkinson y Otros Trastornos del Movimiento, INIBIC ( Instituto de Investigación Biomédica de A Coruña), 15006 A Coruña, Spain; 3 Hospital San Rafael, 15006 A Coruña, Spain; 4 Fundación Degen, 15006 A Coruña, Spain; 5 Networking Research Center on Neurodegenerative Diseases (CIBERNED), Instituto de Salud Carlos III, 28029 Madrid, Spain; 6 Complejo Hospitalario de Cartagena, 30202 Murcia, Spain; 7 Complejo Hospitalario Universitario de Ourense (CHUO), 32005 Ourense, Spain; 8 Complejo Hospitalario Universitario de Pontevedra (CHOP), 36164 Pontevedra, Spain; 9 Complejo Hospitalario Universitario de Vigo (CHUVI), 36312 Vigo, Spain; 10 Hospital de Povisa, 36211 Vigo, Spain

**Keywords:** Parkinson’s disease, inhaled levodopa, OFF episodes, non-motor fluctuations, motor fluctuations, temporal dynamics

## Abstract

Background/Objectives: OFF episodes in Parkinson’s disease (PD) include motor and non-motor manifestations, but their resolution after on-demand therapy may not be synchronous. We examined within-patient temporal dynamics of motor and non-motor responses after inhaled levodopa. Methods: This post hoc analysis used data from the prospective, multicenter, open-label INLEVO-LIFE PD study. At week 12, UPDRS-III and 10 OFF-related non-motor symptoms (NMSs) were assessed in OFF and 30, 60, and 90 min after inhaled levodopa. The principal post hoc outcome was the proportion of maximum early (30–60 min) benefit retained at 90 min for motor versus non-motor outcomes. Individual trajectories were also examined descriptively. Results: Thirty-four participants had complete serial assessments. Mean UPDRS-III scores were 25.91, 11.21, 12.00, and 16.00 at OFF, 30, 60, and 90 min, respectively (*p* < 0.0001); corresponding NMSS-OFF/ON total scores were 6.65, 2.09, 1.91, and 2.71 (*p* < 0.0001). Among 28 participants with early improvement in both outcomes, median benefit retained at 90 min was 67.1% for motor symptoms versus 100% for NMSs (*p* = 0.0011). Motor and non-motor improvement magnitudes were not significantly correlated at any time point. Individual trajectories showed marked interindividual heterogeneity in the timing and persistence of motor and non-motor responses. Conclusions: Motor and non-motor OFF manifestations improved rapidly after inhaled levodopa but showed partially dissociated temporal trajectories, challenging the assumption that OFF resolution is necessarily synchronous across symptom dimensions. Greater relative persistence of non-motor benefit at 90 min is hypothesis-generating and requires confirmation using validated NMF instruments and controlled designs.

## 1. Introduction

Parkinson’s disease (PD) is characterized by motor and non-motor manifestations that become increasingly complex as the disease progresses. Long-term dopaminergic treatment is frequently complicated by OFF episodes, during which symptoms re-emerge or worsen as the effect of a levodopa dose wanes [1,2,3,4]. Although OFF has traditionally been defined by motor deterioration, non-motor fluctuations (NMFs) are common, clinically relevant, and may substantially affect quality of life [5,6,7,8,9].

NMFs include neuropsychiatric, cognitive, sensory, autonomic, and other manifestations. Their timing and severity do not necessarily parallel motor fluctuations, and recent work has emphasized the need for dedicated ON/OFF assessment rather than assuming that motor state is an adequate surrogate for non-motor state [10,11,12]. This distinction is relevant when evaluating rescue therapies because the apparent onset, magnitude, and persistence of benefit may depend on the dimension of the OFF episode being measured.

Inhaled levodopa is an on-demand therapy designed to provide rapid levodopa delivery through the pulmonary route and bypass delayed or variable gastrointestinal absorption [13]. Randomized trials, systematic reviews, pharmacokinetic studies, long-term safety studies, and clinical development analyses have established rapid motor benefit and supported its use for intermittent OFF episodes [14,15,16,17,18,19,20,21].

The prospective INLEVO-LIFE PD study extended this evidence by evaluating health-related quality of life and OFF-related non-motor symptoms during 12 weeks of inhaled levodopa use. The parent publication reported improvement in PDQ-39, daily OFF time, and exploratory acute motor and non-motor assessments [22]. However, whether motor and non-motor manifestations evolve in parallel after rescue treatment was not the primary focus of that study. We therefore explored whether resolution of an OFF episode after inhaled levodopa is synchronous across motor and non-motor dimensions or whether these components may follow partially independent temporal trajectories.

## 2. Materials and Methods

### 2.1. Study Design and Participants

This was a post hoc analysis of INLEVO-LIFE PD, a prospective, multicenter, open-label study conducted at five centers in Spain. The parent study enrolled 40 people with PD treated with inhaled levodopa and followed them for 12 ± 2 weeks; 34 participants remained on treatment and completed follow-up. Full eligibility criteria, study procedures, ethics approval, and safety and effectiveness assessments are reported in the parent publication [22]. The present analysis used the de-identified study database supplied for secondary analysis.

The study was not designed to establish a causal treatment effect on the relative duration of motor versus non-motor improvement. The present comparisons are within-person descriptions of observed scores after a single rescue administration rather than a pharmacodynamic comparison between two validated and interchangeable outcomes.

### 2.2. Acute Motor and Non-Motor Assessment

At the 12-week visit, participants were evaluated during an OFF episode immediately before inhaled levodopa and at 30, 60, and 90 min after administration. Motor severity was assessed with UPDRS Part III [23]. Ten OFF-related NMSs were assessed with the study-specific NMSS-OFF/ON: pain, bad mood, anxiety, fatigue, dizziness, mind dullness, akathisia, dysphagia, bladder urgency, and sweating. Each item was rated by the neurologist at that precise moment on a 5-point ordinal severity scale: 0 = none, 1 = mild, 2 = moderate, 3 = severe, and 4 = very severe. The NMSS-OFF/ON total score was the sum of the 10 items and therefore ranged from 0 to 40, with higher scores indicating greater acute NMS burden [22]. This instrument was specifically designed for the parent study and has not been formally validated; analyses involving its total score and individual items were therefore considered exploratory. The levodopa equivalent daily dose was calculated using established conversion factors [24].

The ten items cover a pragmatic range of symptoms potentially experienced during OFF episodes, spanning affective, cognitive, sensory, autonomic and other domains. Their inclusion in the parent protocol should not be interpreted as evidence that a formal consensus-based item-selection or psychometric validation process was undertaken; the original documentation available for this analysis does not establish such a process. The instrument was used because these repeated symptom-level observations were collected at all four time points and enabled a matched exploratory analysis alongside the motor examination.

### 2.3. Post Hoc Outcomes

The principal post hoc outcome was relative persistence of the acute response at 90 min. For each outcome, maximum early benefit was defined as the larger improvement from OFF observed at 30 or 60 min. In participants showing an early improvement in both motor and NMS outcomes, persistence was calculated as: [(OFF score − 90-min score)/maximum early improvement] × 100. Secondary analyses examined (1) correlations between motor and NMS improvement at each time point; (2) trajectories of post hoc UPDRS-III motor components, defined as tremor (items 20–21), rigidity (item 22), bradykinesia (items 23–26 and 31), and axial manifestations (items 18–19 and 27–30); (3) trajectories of each of the 10 NMSs; (4) an exploratory responder definition of ≥30% improvement from OFF; and (5) descriptive visualization of individual motor and non-motor trajectories across OFF, 30, 60, and 90 min. Individual trajectories were displayed using raw scores to avoid unstable percentage normalization in participants with very low OFF-state scores. No post hoc phenotypic subgroups were formally defined.

For this analysis, early improvement required a strictly positive reduction from the OFF score at either 30 or 60 min, separately for each outcome. Participants entered the paired persistence comparison only if both outcomes had a positive maximum early improvement, thereby avoiding a zero or negative denominator. The best response across the two early visits was selected to accommodate variation in the time of peak observed benefit, but this data-dependent choice can exaggerate the denominator through measurement noise and regression to the mean. Values may exceed 100% when the 90-min score is better than the best observed early score; negative values indicate worsening beyond the initial OFF score. Percent persistence therefore represents a descriptive within-scale ratio, not a validated cross-scale equivalence of clinical benefit.

### 2.4. Statistical Analysis

Analyses were performed on available complete paired observations for the acute OFF–30–60–90 min assessment. Continuous data are summarized as mean ± SD or median and interquartile range, as appropriate. Overall within-participant temporal changes were tested using Friedman tests. The paired comparison of motor versus non-motor persistence at 90 min used the Wilcoxon signed-rank test. Associations between the magnitude of motor and non-motor improvement were assessed with Spearman rank correlations. For the 10 individual NMS temporal tests, Holm’s correction controlled the family-wise error rate. The ≥30% responder analyses were supportive, and no validated minimal clinically important difference was assumed for the study-specific NMSS-OFF/ON total score. Two-sided *p* < 0.05 was considered statistically significant. Given the post hoc, exploratory nature and modest sample size, effect patterns and consistency were emphasized over isolated *p* values. Statistical calculations were performed in Python 3.13.5 using pandas 2.2.3, SciPy 1.17.0, and statsmodels 0.14.6.

The persistence comparison is conditional on early improvement in both dimensions, whereas descriptive trajectories include all 34 complete cases and the non-motor responder analysis includes only the 28 participants with at least one symptom present during OFF. These analytic populations address different questions and should not be compared as though they had identical eligibility. No imputation was applied to missing acute observations. Because individual-level source data were not supplied for the present revision, additional confidence intervals and effect-size calculations have not been introduced without independent reproducibility checks.

## 3. Results

### 3.1. Analysis Population

Thirty-four participants had complete UPDRS-III and all 10 NMSS-OFF/ON item assessments at OFF, 30, 60, and 90 min. Their mean age was 62.0 ± 8.9 years, 20 (58.8%) were men, mean PD duration was 8.15 ± 4.72 years, and mean levodopa equivalent daily dose was 1058 ± 404 mg/day. Baseline daily OFF time was 4.21 ± 2.42 h and mean baseline PDQ-39 total score was 43.59 ± 24.24 (Table 1). Six of the 40 participants enrolled in the parent study did not contribute complete week-12 serial data. Consequently, the acute results reflect a completer population and may not represent participants who discontinued treatment or were unable to complete the assessment. The limited sample and the conditional nature of the post hoc outcomes restrict generalizability.

### 3.2. Overall Temporal Dynamics

**Figure 1:**
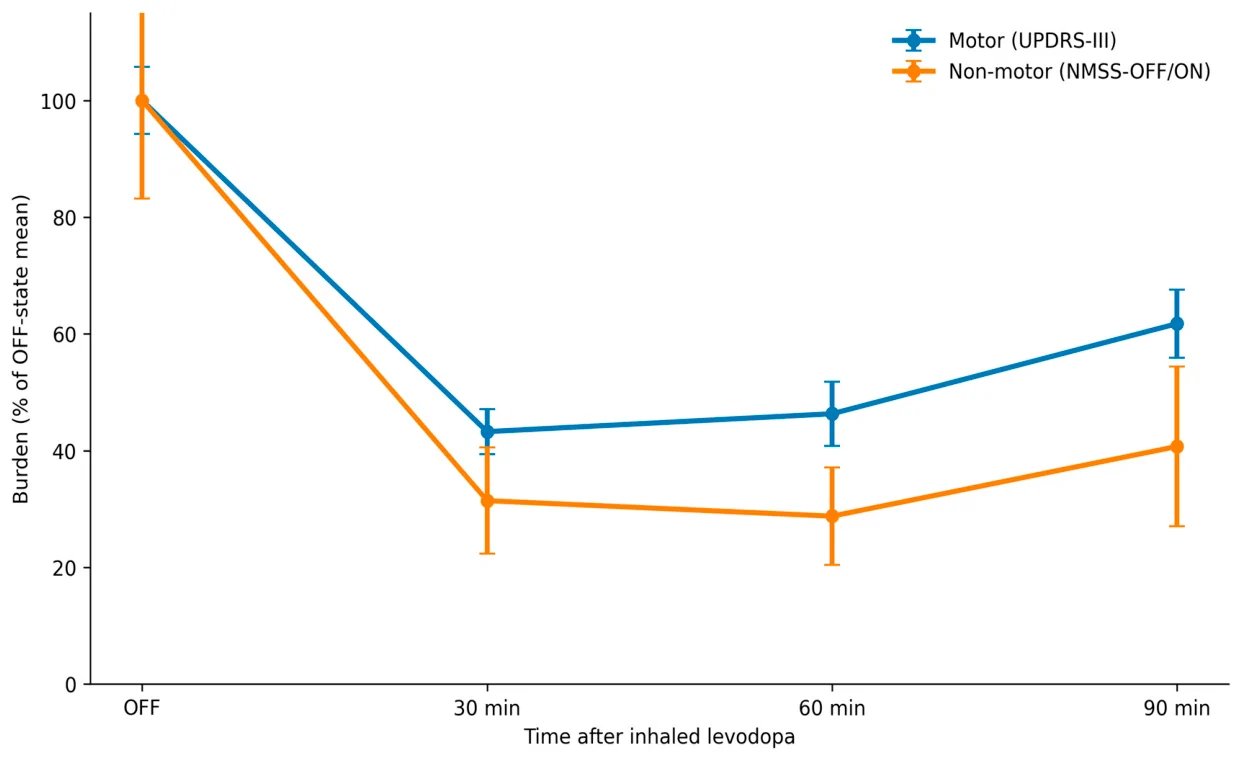
Temporal dynamics of motor and non-motor burden after inhaled levodopa. Values are normalized to the corresponding OFF-state mean; error bars represent standard errors.

Mean UPDRS-III scores decreased from 25.91 ± 8.72 in OFF to 11.21 ± 5.81 at 30 min and 12.00 ± 8.31 at 60 min, followed by partial worsening to 16.00 ± 8.87 at 90 min (Friedman *p* < 0.0001). The NMSS-OFF/ON total score decreased from 6.65 ± 6.53 in OFF to 2.09 ± 3.53, 1.91 ± 3.25, and 2.71 ± 5.31 at 30, 60, and 90 min, respectively (*p* = 3.93 × 10^−12^). The numerical trajectories are summarized in Table 2, and their normalized temporal profiles are shown in Figure 1.

### 3.3. Motor-Domain Trajectories

**Figure 2:**
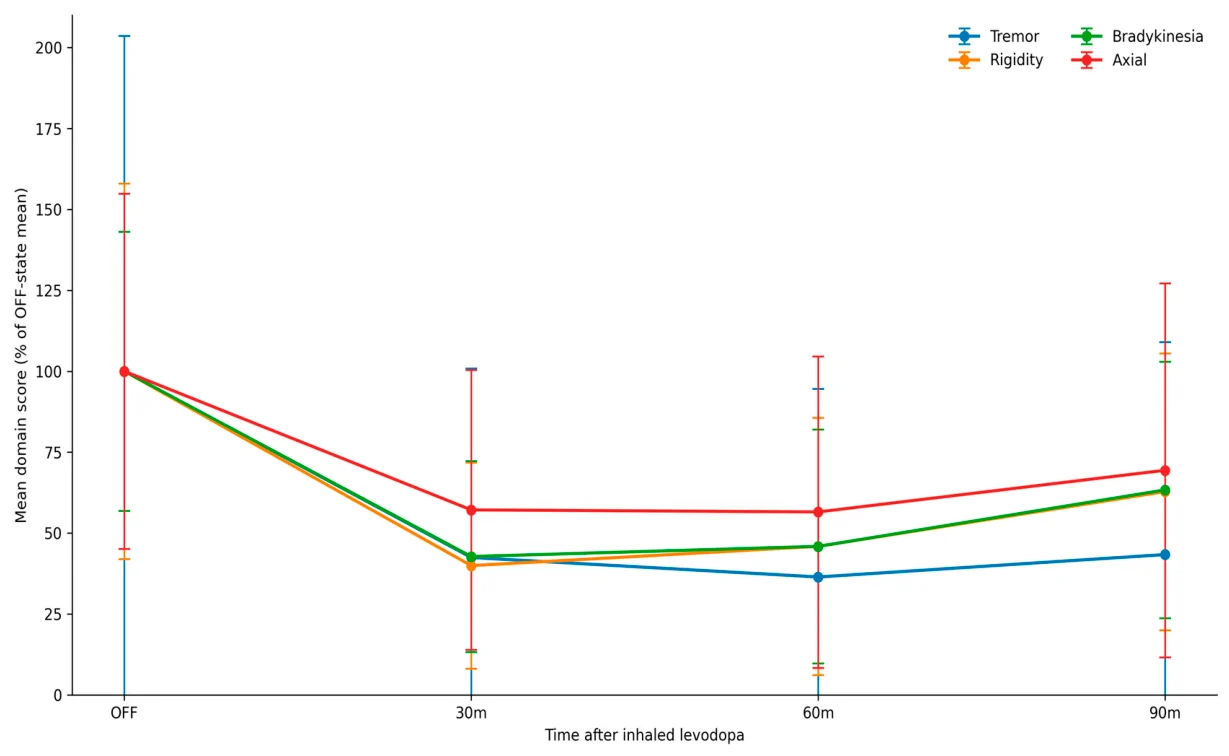
Temporal trajectories of post hoc UPDRS-III motor components, normalized to each component’s OFF-state mean. Components were defined as tremor (items 20–21), rigidity (item 22), bradykinesia (items 23–26 and 31), and axial manifestations (items 18–19 and 27–30). Data are presented as mean ± SD.

All four motor components improved over time. Mean OFF/30/60/90 min scores were 3.32/1.41/1.21/1.44 for tremor, 5.38/2.15/2.47/3.38 for rigidity, 12.44/5.32/5.71/7.88 for bradykinesia, and 4.74/2.71/2.68/3.29 for axial manifestations. Overall temporal effects were significant for each domain (all *p* < 0.0001). Tremor showed particularly sustained improvement through 90 min, whereas rigidity and bradykinesia showed more evident partial return toward OFF-state severity (Figure 2).

### 3.4. Persistence and Motor–Non-Motor Dissociation

**Figure 3:**
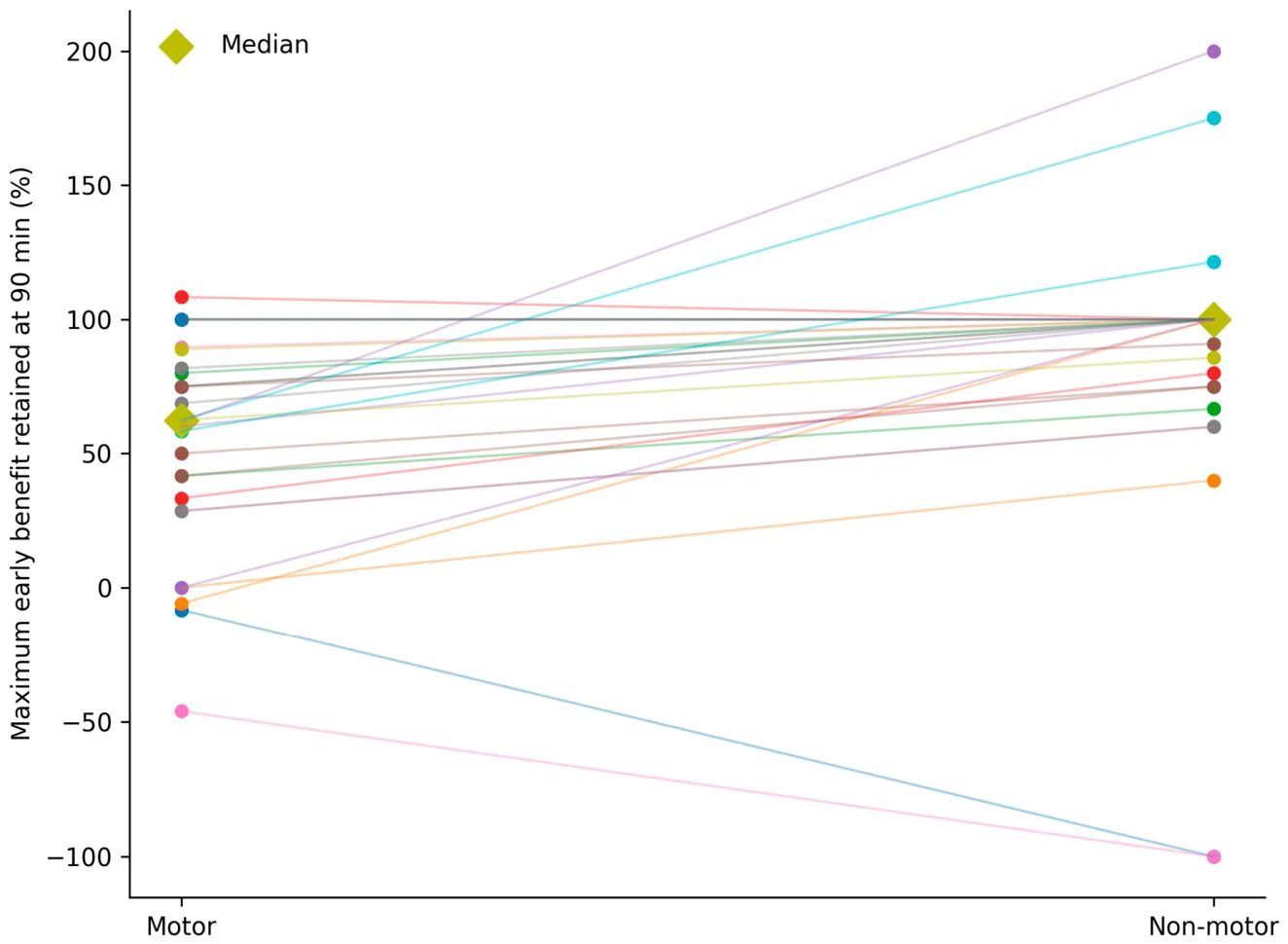
Within-participant comparison of the proportion of maximum early benefit retained at 90 min in motor and non-motor outcomes (N = 28 with early improvement in both).

**Figure 4:**
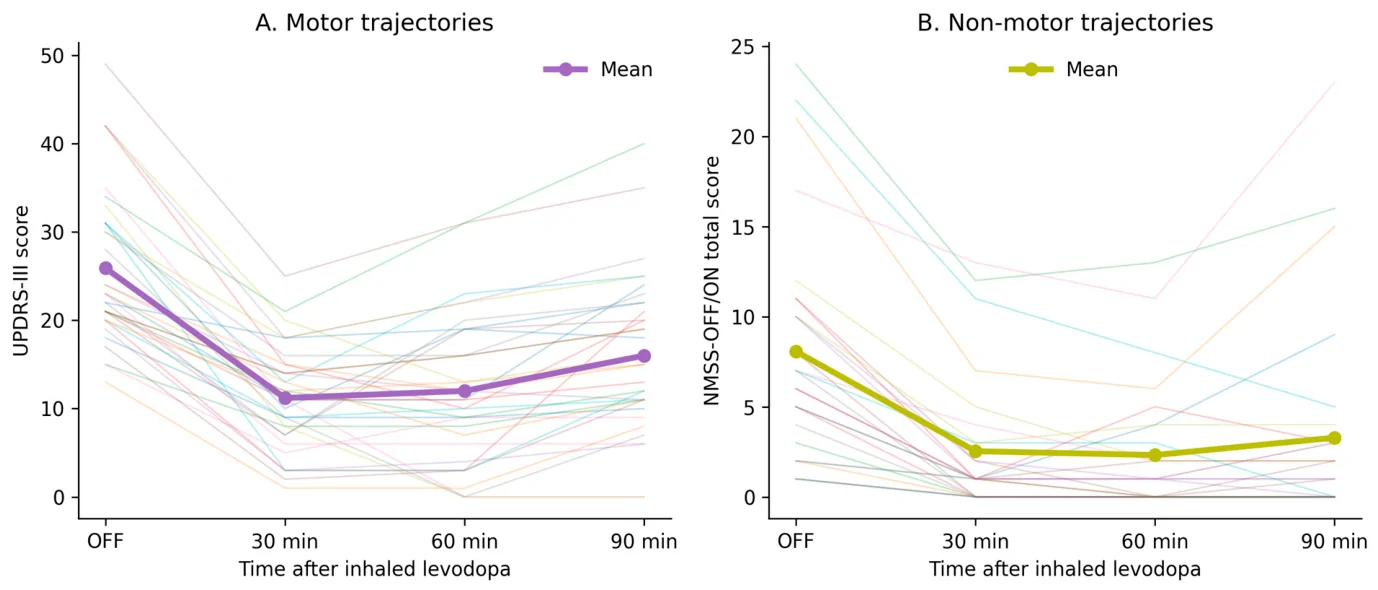
Individual raw-score trajectories of motor burden (UPDRS-III; N = 34) and non-motor burden (NMSS-OFF/ON total score; N = 28 with ≥1 OFF NMS) from OFF to 30, 60, and 90 min after inhaled levodopa. Thin lines represent individual participants, and the thick line represents the group mean. Raw scores are displayed to avoid unstable percentage normalization in participants with very low OFF-state scores. The figure is descriptive and does not define response phenotypes.

Twenty-eight participants showed an evaluable early improvement in both motor and NMSS-OFF/ON outcomes. The median proportion of maximum early benefit retained at 90 min was 67.1% (IQR 32.1–81.8) for motor symptoms and 100% (IQR 75.0–100) for NMSs (paired Wilcoxon *p* = 0.0011; Figure 3). The magnitude of motor improvement was not significantly correlated with NMS improvement at 30 min (Spearman’s ρ = −0.188, *p* = 0.286), 60 min (ρ = −0.256, *p* = 0.144), or 90 min (ρ = 0.094, *p* = 0.596). Individual raw-score trajectories are shown in Figure 4. Visual inspection showed that motor and non-motor recovery did not follow a uniform within-patient pattern; however, these patterns were not classified into formal subgroups because no prespecified or validated thresholds for such phenotypes were available.

These percentages compare retention of the maximum improvement within each instrument; they do not imply that a one-percentage-point change has equivalent clinical significance on UPDRS-III and the study-specific symptom score. The persistence comparison excludes six complete cases without positive early improvement in both measures. The median non-motor value of 100% should not be read as indicating that every individual retained all benefit. Of the 34 complete cases, 28 (82.4%) showed positive early improvement in both domains, while six (17.6%) improved only in the motor domain. All six had an NMSS-OFF/ON score of zero in the OFF state, precluding measurable non-motor improvement; none showed non-motor-only improvement or improvement in neither domain.

### 3.5. Individual Non-Motor Symptoms

After Holm’s correction across the 10 NMSS-OFF/ON items, significant temporal effects remained for fatigue (adjusted *p* = 1.31 × 10^−8^), bad mood (2.03 × 10^−6^), mind dullness (2.94 × 10^−5^), sweating (0.00097), anxiety (0.00295), and dizziness (0.00309). Pain showed a nominal temporal effect (unadjusted *p* = 0.031) but did not remain significant after correction (adjusted *p* = 0.122). Akathisia, dysphagia, and bladder urgency were not significant after multiplicity adjustment (Table S1).

### 3.6. Exploratory Responder Analysis

Using a supportive ≥30% improvement threshold, motor responder proportions were 33/34 (97.1%) at 30 min, 26/34 (76.5%) at 60 min, and 20/34 (58.8%) at 90 min. Among the 28 participants with at least one NMS present in OFF, NMS responder proportions were 27/28 (96.4%), 27/28 (96.4%), and 25/28 (89.3%), respectively (Table S2). The corresponding motor–non-motor correlation coefficients are summarized in Table S3. These results should not be interpreted as validated clinical responder rates. The responder denominators differ by design (motor: 34; non-motor: 28 with at least one OFF symptom). Moreover, the 30% cut-off has no established minimal clinically important difference for NMSS-OFF/ON. The apparent contrast in responder proportions across time therefore cannot establish comparative durability or clinical superiority of non-motor response.

## 4. Discussion

This post hoc analysis suggests that the acute motor and non-motor components of an OFF episode do not follow identical trajectories after inhaled levodopa. Both improved markedly within 30–60 min, but the relative non-motor benefit was more sustained at 90 min, and the magnitude of motor improvement was poorly coupled to the magnitude of NMS improvement within individual participants. The key observation is therefore not simply that NMSs improve after inhaled levodopa—a finding already described in the parent INLEVO-LIFE PD report—but that resolution of an OFF episode may be asynchronous across symptom dimensions, with motor and non-motor responses showing partial temporal dissociation. Importantly, the central comparison concerns the time course of two recorded measurements, not two biologically isolated treatment effects. A non-significant correlation between change scores is not evidence of statistical equivalence or independence, particularly in a modest sample with restricted non-motor score variability.

The individual trajectories reinforce this group-level observation by showing marked heterogeneity in both the magnitude and persistence of response. Some participants showed broadly parallel motor and non-motor improvement, whereas others showed visibly discordant trajectories. These descriptive patterns should not be interpreted as validated response phenotypes or subgroups; identifying reproducible motor–non-motor response phenotypes would require larger, prospectively designed cohorts and prespecified classification criteria. This finding is clinically plausible in the context of the prior NMF literature. OFF-related anxiety, low mood, fatigue, cognitive slowing, pain, sweating, and other autonomic manifestations are well recognized, but NMFs do not always coincide precisely with motor state transitions [5,6,7,8,9,10,11,12]. Acute levodopa challenge studies further show that non-motor responsiveness is heterogeneous across symptoms and patients [25]. Pain, in particular, may fluctuate in association with both motor and other non-motor manifestations [26]. One possible biological explanation, requiring direct testing, is that different symptoms have differing dependence on dopaminergic signaling and on striatal versus extrastriatal circuits. Motor signs prominently engage basal ganglia motor networks, whereas mood, anxiety and cognitive complaints may involve limbic and cortical systems, and autonomic symptoms involve additional peripheral and central regulatory networks. Such circuitry could plausibly produce different thresholds or response kinetics after dopaminergic stimulation. Nevertheless, the study measured neither regional neurotransmission nor network activity; differences in symptom reporting, observation and scoring remain equally plausible explanations. These possibilities must not be mistaken for demonstrated mechanistic dissociation [7,8,25,27].

In the present dataset, fatigue, bad mood, anxiety, dizziness, mind dullness, and sweating retained significant temporal effects after multiplicity correction. This symptom-level heterogeneity is consistent with the contemporary NMF literature and supports assessment of individual non-motor domains rather than reliance on a single global motor-state label [8,10,11,12,25,26]. Dysphagia, as well as pain, akathisia and bladder urgency, did not show a statistically significant temporal change after multiplicity correction. This should not be interpreted as evidence that swallowing-related OFF symptoms are unresponsive to levodopa. The number of participants reporting dysphagia, limited baseline severity, the short observation window and the use of a single ordinal item may all reduce sensitivity to detect change. The same would apply to the other non-motor symptoms without statistical significance. Because dysphagia has important safety implications, targeted assessments with validated swallowing measures would be required before drawing treatment-related conclusions [7,8,25].

The motor trajectory was consistent with the established rapid efficacy of inhaled levodopa during OFF episodes [14,15,16,17,18,19,20,21]. The domain analysis further suggested that durability of response may differ across motor manifestations, with tremor remaining relatively suppressed at 90 min and rigidity and bradykinesia showing more evident partial return toward OFF-state severity. These domain findings are exploratory because the grouped UPDRS-III components were defined post hoc.

The greater relative persistence of NMS benefit observed in this exploratory analysis should not be interpreted as evidence that inhaled levodopa has a longer pharmacological effect on non-motor than motor pathways. Several alternative explanations are possible, including differences in measurement scale properties, symptom thresholds, floor effects, regression to the mean, and non-dopaminergic contributions to individual NMSs. Moreover, the absence of correlation between motor and NMS response does not establish mechanistic independence. Rather, these findings support prospective investigation using validated NMF instruments and denser temporal sampling.

A particular concern is the low baseline mean NMSS-OFF/ON total (6.65 out of 40), with substantial dispersion (SD 6.53). Participants with absent or minimal OFF symptoms have little room for improvement, and those with only one or two mildly affected items can reach a score of zero after a small absolute change. Once at zero, the total cannot improve further, potentially creating an apparent plateau at 30, 60 and 90 min. The ratio of 90 min to maximum early improvement is also sensitive to small denominators: a change of only one ordinal point can shift the calculated percentage markedly. Conditioning the paired analysis on positive early change does not remove this measurement problem and may amplify selection effects. A future prospective study should report baseline severity strata, item-level distributions and absolute changes alongside normalized trajectories and should prespecify sensitivity analyses excluding participants with very low baseline symptom burden [10,11,12].

From a treatment perspective, these observations complement the established motor efficacy of inhaled levodopa and the broader rationale for gastrointestinal-bypassing on-demand therapies [2,3,13,14,15,16,17,18,19,20,21]. Recent post-commercialization experience has supported rapid perceived improvement in OFF symptoms in routine practice [28], while a 2026 network meta-analysis has placed inhaled levodopa within the contemporary evidence base for on-demand OFF treatment [29]. A recent Spanish expert consensus also reported high agreement on the utility of inhaled levodopa for rapid treatment of both motor and non-motor fluctuations [30]. More broadly, current reviews of motor and non-motor fluctuations emphasize that therapeutic assessment should capture the multidimensional burden of OFF rather than motor function alone [31].

This analysis has important limitations. INLEVO-LIFE PD was open-label and lacked a placebo or active comparator. The present analyses were post hoc and involved only 34 complete cases, limiting precision and precluding complex multivariable modeling. The acute 10-item NMSS-OFF/ON used a 0–4 ordinal severity rating for each symptom and was specifically developed for the parent study; it has not been formally validated. Validated instruments such as the Non-Motor Fluctuation Assessment Questionnaire are now available for characterizing NMFs [12]. The total score gives equal weight to symptoms with potentially different clinical meaning, and floor effects are possible when symptoms are absent or mild in OFF. The 30 min sampling interval cannot establish exact onset or offset of benefit. Multiple secondary analyses increase the risk of false-positive findings, although Holm’s correction was applied to individual NMS comparisons. Persistence was expressed relative to each participant’s maximum early improvement; this is useful for comparing trajectories but is not a validated clinical endpoint. The six participants excluded from the paired persistence comparison had no non-motor symptom burden in the OFF state, indicating a floor effect rather than demonstrable motor–non-motor response dissociation. Thus, the persistence comparison applies specifically to participants with measurable early improvement in both domains. Finally, the UPDRS-III and NMSS-OFF/ON differ in item content, range, scaling, responsiveness and measurement error. Normalization by maximum early change does not make them psychometrically comparable. The NMSS-OFF/ON has not undergone testing for reliability, construct validity, responsiveness or clinically important change, and its summed score assumes equal weighting of ten heterogeneous manifestations. The present analysis therefore cannot disentangle clinical asynchrony from instrument-related effects. In addition, open-label assessment may be affected by expectation, observation and reporting biases. Without a control condition or pharmacokinetic sampling, the observed score patterns cannot establish treatment-specific mechanisms or differential duration of drug action [10,11,12].

Despite these limitations, the within-patient design and simultaneous serial motor and non-motor measurements provide a distinctive view of OFF-episode recovery after rescue levodopa. The findings generate the hypothesis that clinically relevant NMFs may persist or resolve on a different time course from conventional motor manifestations, which could influence how patients and clinicians judge the success and duration of on-demand treatment. Future confirmation should use a prospectively defined motor and non-motor assessment battery, validated non-motor fluctuation instruments, shorter and more frequent post-dose intervals, and adequate representation of participants with a broad range of baseline non-motor burden. Reporting absolute and relative trajectories, confidence intervals for paired contrasts, and sensitivity analyses by baseline symptom severity would help distinguish measurement effects from reproducible clinical phenomena. A controlled design would also help separate levodopa-related changes from expectancy effects and spontaneous fluctuation during the observed OFF episode [12].

## 5. Conclusions

In people with PD experiencing OFF episodes, inhaled levodopa was followed by rapid improvement in both motor manifestations and OFF-related NMS. Their subsequent trajectories were only partially coupled, suggesting that resolution of an OFF episode should not necessarily be conceptualized as a synchronous motor–non-motor phenomenon: non-motor benefit showed greater relative persistence at 90 min, while individual motor and non-motor response magnitudes were not significantly correlated. These exploratory observations support prospective studies specifically designed to characterize the temporal dynamics of NMFs using validated measures and more frequent sampling. The observed temporal differences between the UPDRS-III and study-specific NMSS-OFF/ON scores are exploratory and do not demonstrate that inhaled levodopa exerts a longer pharmacological action on non-motor than motor symptoms. Interpretation should account for the unvalidated non-motor instrument, its low OFF-state burden and potential floor effects.

## Figures and Tables

**Table 1:** Characteristics of the complete-case post hoc analysis population (N = 34).

Characteristic	Value
Age, years	62.0 ± 8.9
Male sex, n (%)	20 (58.8)
Parkinson’s disease duration, years	8.15 ± 4.72
LEDD, mg/day	1058 ± 404
Daily OFF time at baseline, h	4.21 ± 2.42
Hoehn & Yahr stage ON, median	2
PDQ-39 total score at baseline	43.59 ± 24.24
MDS-NMS total score at baseline	132.44 ± 88.82

**Table 2:** Acute motor and non-motor trajectories at the 12-week assessment. Values are mean ± SD. Overall *p* values are from Friedman tests.

Outcome	OFF	30 min	60 min	90 min	Overall *p*
UPDRS-III	25.91 ± 8.72	11.21 ± 5.81	12.00 ± 8.31	16.00 ± 8.87	<0.0001
NMSS-OFF/ON	6.65 ± 6.53	2.09 ± 3.53	1.91 ± 3.25	2.71 ± 5.31	3.93 × 10^−12^
Tremor	3.32 ± 3.44	1.41 ± 1.94	1.21 ± 1.93	1.44 ± 2.18	1.74 × 10^−10^
Rigidity	5.38 ± 3.12	2.15 ± 1.71	2.47 ± 2.14	3.38 ± 2.30	2.98 × 10^−11^
Bradykinesia	12.44 ± 5.36	5.32 ± 3.67	5.71 ± 4.49	7.88 ± 4.93	1.55 × 10^−12^
Axial	4.74 ± 2.60	2.71 ± 2.05	2.68 ± 2.28	3.29 ± 2.74	2.23 × 10^−10^

## Data Availability

The protocol and the statistical analysis plan are available on request. Deidentified participant data are not available for legal and ethical reasons.

## Supplementary Materials

The following supporting information can be downloaded at: https://www.mdpi.com/article/10.3390/medsci14060680/s1: Table S1, Individual non-motor symptom trajectories and multiplicity-adjusted tests; Table S2, Exploratory ≥ 30% responder rates; Table S3, Motor–non-motor response correlations.
