# Supplementary material for: Differential Temporal Dynamics and Motor–Non-Motor Dissociation After Inhaled Levodopa in Parkinson’s Disease: A Post Hoc Analysis of the INLEVO-LIFE PD Study
Source: Med Sci (Basel). 2026 Oct 17;14(6):680. doi: 10.3390/medsci14060680 (PMC13707020; doi:10.3390/medsci14060680)
Supplement: Supplementary file 1 [file medsci-14-00680-s001.zip › medsci-4621389-supplementary.pdf]

Supplementary Material

Differential Temporal Dynamics and Motor–Non-Motor Dissociation after Inhaled Levodopa in Parkinson's Disease: A Post Hoc Analysis of the INLEVO-LIFE PD Study

Table S1. Individual non-motor symptom trajectories and multiplicity-adjusted temporal tests

Symptom	OFF	30 min	60 min	90 min	Raw p	Holm-adjusted p	Result
Pain	0.65 ± 1.12	0.44 ± 0.96	0.38 ± 0.92	0.35 ± 0.88	0.0305	0.122	Not significant
Bad mood	1.03 ± 1.38	0.24 ± 0.78	0.21 ± 0.59	0.32 ± 0.84	2.26e-07	2.03e-06	Significant
Anxiety	0.85 ± 1.18	0.29 ± 0.63	0.21 ± 0.48	0.53 ± 1.05	0.000492	0.00295	Significant
Fatigue	1.26 ± 1.33	0.35 ± 0.85	0.29 ± 0.68	0.50 ± 0.93	1.31e-09	1.31e-08	Significant
Dizziness	0.56 ± 0.99	0.09 ± 0.29	0.12 ± 0.33	0.21 ± 0.59	0.000618	0.00309	Significant
Mind dullness	0.82 ± 0.97	0.18 ± 0.52	0.32 ± 0.68	0.29 ± 0.68	3.67e-06	2.94e-05	Significant
Akathisia	0.44 ± 0.89	0.21 ± 0.48	0.15 ± 0.36	0.18 ± 0.52	0.212	0.456	Not significant
Dysphagia	0.15 ± 0.50	0.09 ± 0.38	0.03 ± 0.17	0.06 ± 0.34	0.152	0.456	Not significant
Bladder urgency	0.29 ± 0.76	0.09 ± 0.29	0.12 ± 0.33	0.09 ± 0.38	0.156	0.456	Not significant
Sweating	0.59 ± 0.96	0.12 ± 0.41	0.09 ± 0.38	0.18 ± 0.52	0.000138	0.000969	Significant

Values are mean ± SD NMSS-OFF/ON item scores (0 = none, 1 = mild, 2 = moderate, 3 = severe, 4 = very severe) in the 34 complete cases. Raw p values are from Friedman tests; Holm's adjustment was applied across the 10 individual NMS temporal tests.

Table S2. Exploratory ≥30% responder rates

Outcome	30 min	60 min	90 min
Motor response (UPDRS-III), n=34	33/34 (97.1%)	26/34 (76.5%)	20/34 (58.8%)
NMSS-OFF/ON response among participants with ≥1 OFF NMS, n=28	27/28 (96.4%)	27/28 (96.4%)	25/28 (89.3%)

Responder was defined post hoc as a ≥30% improvement from OFF. This threshold is exploratory and is not a validated minimal clinically important difference for the study-specific NMSS-OFF/ON.

Table S3. Correlation between motor and non-motor improvement

Time point	Spearman ρ	p value	Interpretation
30 min	−0.188	0.286	No significant correlation
60 min	−0.256	0.144	No significant correlation
90 min	0.094	0.596	No significant correlation

Improvement scores were calculated relative to OFF. These analyses support partial motor–non-motor dissociation but do not establish mechanistic independence.

Supplementary statistical note

The acute complete-case cohort comprised 34 participants. For the paired persistence analysis, 28 participants had evaluable initial improvement in both motor and non-motor outcomes. Maximum early benefit was defined as the larger improvement from OFF to 30 min or from OFF to 60 min. Persistence at 90 min was calculated as [(OFF - 90 min)/(maximum early improvement)] × 100. Median persistence was 62.2% (IQR 32.1-80.5) for motor symptoms and 100% (IQR 75-100) for non-motor symptoms; paired Wilcoxon's p=0.0011.
